# Engineering Immunity: Current Progress and Future Directions of CAR-T Cell Therapy

**DOI:** 10.3390/ijms27020909

**Published:** 2026-01-16

**Authors:** Mouldy Sioud, Nicholas Paul Casey

**Affiliations:** Division of Cancer Medicine, Department of Cancer Immunology, Oslo University Hospital—Rdiumhospitalet, Ullernchausseen 70, 0379 Oslo, Norway

**Keywords:** chimeric antigen receptor, messenger RNA, lipid nanoparticles, tumor microenvironment, T cell therapy, cancer, autoimmunity

## Abstract

Chimeric antigen receptor (CAR)-T cell therapy has emerged as a transformative form of immunotherapy, enabling the precise engineering of T cells to recognize and eliminate pathogenic cells. In hematologic malignancies, CAR-T cells targeting CD19 or B cell maturation antigens have achieved remarkable remission rates and durable responses in patients with otherwise refractory disease. Despite these successes, extending CAR-T cell therapy to solid tumors remains challenging due to antigen heterogeneity, poor T cell infiltration, and the immunosuppressive tumor microenvironment (TME). Beyond oncology, CAR-T cell therapy has also shown promise in autoimmune diseases, where early clinical studies suggest that B cell-directed CAR-T cells can induce sustained remission in conditions such as systemic lupus erythematosus. This review highlights advances in CAR-T cell engineering, including DNA- and mRNA-based platforms for ex vivo and in vivo programming, and discusses emerging strategies to enhance CAR-T cell trafficking, persistence, and resistance to TME.

## 1. Introduction

Chimeric antigen receptor (CAR)-T cell therapy is an innovative form of immunotherapy in which a patient’s T cells are genetically engineered to recognize and eliminate target cells [1] By equipping cells with synthetic receptors that bind to tumor-associated antigens, CAR-T cell therapy enables potent and specific tumor targeting that bypasses traditional antigen presentation pathways. In hematologic malignancies, this approach has achieved remarkable success, leading to the approval of several CAR products. For instance, CD19-directed CAR-T cell therapy has transformed the treatment of relapsed or refractory (R/R) B cell acute lymphoblastic leukemia (B-ALL), especially in pediatric and young-adult populations, with meta-analyses reporting a pooled overall response rate (ORR) of about 85% and a complete remission rate (CRR) of 82% [2,3]. Long-term follow-up indicated durable remissions in several patients, with ORR and CRR reaching 62% [4], and similarly high initial responses reported in R/R B cell non-Hodgkin lymphoma [5]. B cell maturation antigen (BCMA)-targeted CAR-T cells have also shown strong efficacy in R/R multiple myeloma. Ide-cel (Abecma), which incorporates a single BCMA-targeting scFv domain, achieved an ORR of 73% and a CR rate of 33% in the KarMMa trial, with a median progression-free survival of 8.8 months [6]. In the KarMMa-3 trial, ide-cel reached an ORR of approximately 71% and a CR rate of 39%, clearly outperforming standard therapies [7]. Cilta-cel (ciltacabtagene autoleucel) is another CAR-T cell therapy that has improved outcomes for patients with R/R multiple myeloma. In heavily pretreated patients, it has demonstrated durable responses with a tolerable safety profile [8,9]. Cilta-cel features a unique CAR design that incorporates two camelid single-domain antibody fragments (VHHs) targeting two distinct epitopes on BCMA. Such design may increase avidity and enhance efficacy at a relatively low single dose. Overall, these clinical data highlight the robust antitumor efficacy of CD19- and BCMA-directed CAR-T cell therapies. Nevertheless, comprehensive assessment of long-term efficacy and safety, together with ongoing patient monitoring, remains essential.

For hematologic malignancies, intravenous delivery of CAR-T cells is highly effective because malignant cells circulate in the blood and bone marrow, making them readily accessible. In contrast, solid tumors remain a major unmet need due to challenges such as the lack of truly tumor-specific antigens, heterogeneous antigen expression, limited CAR-T cell infiltration and persistence, and the immunosuppressive tumor microenvironment (TME), all of which collectively restrict therapeutic efficacy [10]. To address these limitations, current research focuses on strategies including dual-target CARs, armored CAR-T cells engineered to secrete immune-enhancing factors, and rational combinations with checkpoint inhibitors or other immunotherapies [11]. Beyond oncology, researchers are increasingly exploring this adaptive T cell therapy for non-malignant diseases, particularly autoimmune disorders such as systemic lupus erythematosus (SLE), immune thrombocytopenia, rheumatoid arthritis, and multiple sclerosis [12]. In these conditions, autoreactive B or T cells drive tissue damage. CAR-T cell therapy provides a targeted method to eliminate these pathogenic populations; for instance, CD19-directed CAR-T cells have shown promise in refractory SLE by depleting autoreactive B cells and promoting an immune reset [13]. Unlike broad immunosuppressive therapies, CAR-T cell therapy may offer durable and precise disease control. Here, we present an updated overview of CAR-T cell therapy, focusing on recent advances in design and delivery strategies and the key challenges that remain.

## 2. Evolution of CAR-T Engineering

**Figure 1:**
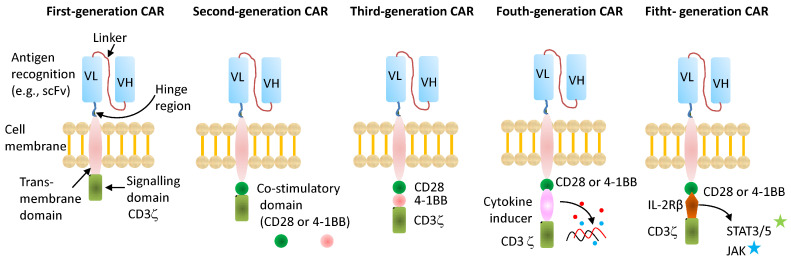
Schematic diagram depicting the structure of first-, second-, third-, fourth-, and fifth-generation chimeric antigen receptors (CARs). First-generation CARs consist of a single-chain variable fragment (scFv) binding domain linked via hinge and transmembrane (TM) domains to an intracellular signaling domain derived from the CD3ζ subunit of the T cell receptor complex. The second-generation CARs have the same elements as the first generation but with the addition of a costimulatory domain usually derived from CD28 or 4-1BB, but may also be derived from ICOS, OX-40, etc. Third-generation CARs include two such costimulatory domains in tandem, in addition to CD3ζ. Fourth-generation CARs replace the additional costimulatory domain with a domain encoding for cytokine expression (e.g., IL-12, IL-15). Fifth-generation CARs replace the cytokine inducer domain with a stimulatory domain from a cytokine receptor (e.g., IL-2Rβ).

CARs generally consist of an extracellular antigen-recognition domain, a transmembrane domain, and one or more intracellular signaling domains that activate T cells upon antigen binding (Figure 1). The extracellular domain is typically a single-chain variable fragment (scFv) derived from an antibody specific for the target antigen. Early CAR development used chimeric receptors in which immunoglobulin variable regions were fused to T cell receptor (TCR) α or β chains [14,15], providing antibody-type recognition with TCR signaling. Eshhar et al. introduced the first scFvs fused to the γ or ζ subunits of TCR/CD3, creating a single-chain receptor capable of both antigen recognition and T cell activation [16]. These constructs represent the ‘first generation’ CARs, combining antigen recognition with a single intracellular CD3ζ signaling domain. While these CARs could activate T cells upon antigen engagement, the absence of costimulatory signaling led to limited proliferation, short persistence, and insufficient clinical activity [17]. The second generation of CARs was developed to address these challenges by adding a costimulatory domain, most commonly CD28 or 4-1BB, in tandem with CD3ζ [18,19]. This dual signaling greatly improved T cell survival, proliferation, and antitumor potency, and formed the backbone of the currently approved therapy targeting CD19 or BCMA in hematologic malignancies [20]. In some studies, CD28-costimulated CAR-T cells exhibited higher cytokine release, whereas 4-1BB-costimulated CAR-T cells showed greater persistence [21,22]. Hence, the third-generation CARs was designed to combine the benefits of multiple costimulatory domains into a single domain, such as the inclusion of both CD28 and 4-1BB, in addition to CD3ζ, with the goal of further amplifying activation signals [23].

Although dual-costimulatory CARs can modify T cell expansion and persistence and, these biological differences do not consistently translate into better clinical outcomes [24,25,26,27]. In the most direct human comparison to date, Ramos et al. conducted a first-in-human, within-patient comparison of second-generation (CD28-only) versus third-generation (CD28 + 4-1BB) CD19-specific CAR-T products in patients with R/R B cell non-Hodgkin lymphoma. In this trial, 16 patients received both products simultaneously, enabling paired tracking of each CAR population in vivo. Third-generation CAR-T cells demonstrated significantly higher CAR expression and longer persistence in peripheral blood than their second-generation counterparts [24]. These differences were most pronounced in patients with low disease burden and depleted normal B cells, in whom second-generation CARs exhibited limited expansion. Clinically, 6 of 11 patients with measurable disease achieved objective responses and the toxicity profile was manageable. The study was small and not statistically powered to demonstrate superiority for durable response rates, progression-free survival, or overall survival [24]. More recent Phase 1/2 data in R/R chronic lymphocytic leukemia also support the feasibility and safety of third-generation CD19 CAR-T cells with encouraging early efficacy, including a high complete response rate and undetectable minimal residual disease in a majority of the patients-. However, the results are derived from uncontrolled, dose-escalation settings without direct comparison to a second-generation CAR [27]. Notably, the outcomes appear comparable to those observed in some clinical trials employing second-generation CAR-T cells [28,29]. Taken together, the available data suggest that CAR-T cell generation alone (second versus third) does not reliably predict clinical performance. Rather, patient outcomes are influenced by multiple factors, including construct design, disease biology, manufacturing variables, and clinical considerations [28,29]. Constructs incorporating both CD28 and 4-1BB costimulatory domains may offer a balance of rapid proliferative signaling and enhanced long-term persistence. larger controlled studies are required to determine whether these attributes translate into consistent clinical benefit across diverse patient populations.

A major advancement in CAR-T cell therapy came with the development of fourth-generation CARs, also known as “armored” CARs or TRUCKs (T cells Redirected for Universal Cytokine Killing). These next-generation constructs are engineered to include inducible cytokine expression cassettes, such as interleukin (IL)-7, IL-12, or IL-15, which are selectively produced upon CAR activation to enhance T cell persistence, proliferation, and antitumor activity [30]. A notable example is the first-in-human clinical trial of a BCMA-targeted, fourth-generation CAR-T cell engineered to secrete IL-7 and CCL19 in patients with R/R multiple myeloma (NCT03778346). This product demonstrated superior T cell survival, proliferation, migration, and immune cell infiltration, translating into greater therapeutic efficacy than that of conventional CAR-T therapies [31]. Recently, fifth-generation CARs have been developed to incorporate cytokine receptor signaling domains, such as truncated IL-2Rβ with STAT3/5-binding motifs, directly into the CAR construct [32] (Figure 1). This design aims to mimic the natural synergy between the TCR and cytokine receptor pathways, promoting more physiological activation and sustained T cell proliferation. Fourth- and fifth-generation CARs share overlapping conceptual and functional features, both aiming to integrate cytokines, immune-modulatory ligands, or safety switches to remodel the TME, enhance resistance to exhaustion, and mitigate toxicity [33], as discussed in Section 7.

Alongside these signaling innovations, CAR architecture efforts have shifted toward refining the antigen-binding domains. Replacing traditional scFv domains with alternative or synthetic binding scaffolds, such as nanobodies (VHH/VNARs), designed ankyrin repeat proteins (DARPins), and other computationally engineered binders, has been shown to increase stability, reduce immunogenicity, and improve antigen accessibility, particularly in solid tumors where steric hindrance limits conventional scFv-based CARs [8,34,35]. Emerging modular “plug-and-play” CAR platforms further enable the flexible exchange of binding modules and precise control of antigen specificity [36]. A landmark example, CAR-T-ddBCMA, which uses a 73-amino-acid synthetic d-domain instead of a scFv, demonstrated potent activity and acceptable safety in R/R multiple myeloma, validating the clinical feasibility of synthetic binding domains [37]. Moreover, computational and AI-guided design is now driving the creation of de novo CAR binding domains with tunable affinity and minimized off-target reactivity, an important step toward the next generation of precision cellular immunotherapies [38]. Because many models optimize binding more than immune tolerance and antigen processing, new constructs may prove immunogenic, reducing persistence and complicating re-dosing. Hence, rigorous evaluation of immunogenicity, stability, and in vivo performance remains essential.

## 3. Ex Vivo CAR Transfer into T Cells

**Figure 2:**
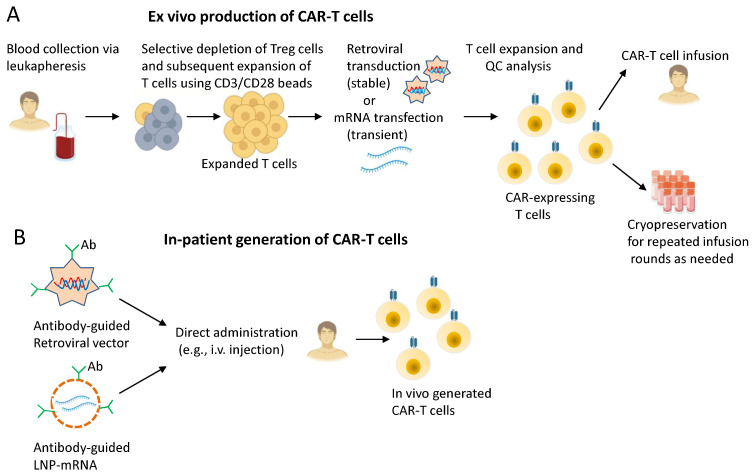
Manufacturing CAR-T cells for adoptive immunotherapy: (**A**) Overview: peripheral blood lymphocytes are isolated from a leukapheresis product by elutriation and subsequently depleted for regulatory T cells before ex vivo expansion in a bioreactor rated for GMP (Good Manufacturing Practice) protocols. The expanded cells are gene-modified to express a CAR by transduction with retroviral vectors or transfection with in vitro transcribed mRNA. Both unmodified and chemically modified mRNA can be used. Following appropriate quality controls (QC), CAR-expressing T cells are re-infused to the patient. (**B**) The direct in vivo delivery of CAR-encoding sequences results in a simpler, shorter, off-the-shelf protocol involving injection of viral or non-viral gene transfer vectors. These are typically (re-)targeted by surface-conjugation of antibodies (e.g., anti-CD8 or anti-CD3 antibodies), with subsequent transformation steps taking place within the target cells themselves (e.g., CD8+ cytotoxic T lymphocytes). Adapted from Almåsback et al. [47] with permission.

The generation of CAR-T cells typically begins with the collection of a patient’s T cells through leukapheresis. These cells are then genetically engineered in the laboratory to express CARs, enabling them to recognize and eliminate specific target cells (e.g., cancer cells) [1]. After sufficient expansion, the modified T cells are administered back to the patient via intravenous infusion (Figure 2A). This infusion is carefully timed, often following a lymphodepleting chemotherapy regimen that enhances CAR-T cell engraftment, expansion, and persistence [1]. Following infusion, patients are closely monitored for adverse effects such as cytokine release syndrome (CRS), neurotoxicity, and infection, ensuring the therapy is administered as safely and effectively as possible [39].

Despite its association with hematologic and infectious toxicities, lymphodepletion remains a fundamental prerequisite for most CAR-T cell therapy protocols. Its omission frequently results in inadequate CAR-T expansion, reduced persistence, and diminished antitumor efficacy [40,41]. Mechanistically, lymphodepletion eliminates immunosuppressive cell populations and reduces competition for homeostatic cytokines such as IL-7 and IL-15, thereby fostering a cytokine-enriched milieu that supports CAR-T proliferation, survival, and early tumor clearance. It may also enhance tumor immunogenicity through transient inflammation within the TME. Although the short-term benefits of lymphodepletion are well established, its long-term effects on CAR-T cell functionality, immune reconstitution, and durable clinical outcomes remain variable across studies [42]. Optimization of conditioning intensity and composition, potentially through biomarker-guided or selective immune-sparing approaches, represents a key area for future investigation.

### Local Administration of CAR-T Cells

Systemic infusion poses inherent challenges, particularly in the treatment of solid tumors, where limited CAR-T cell infiltration and persistence can restrict therapeutic outcomes. To address this challenge, locoregional administration of ex vivo gene-modified CAR-T cells is being explored as an alternative strategy. Locoregional approaches, including intratumoral injection, intrapleural infusion, intracranial infusion, and intra-arterial infusion, are tailored to the tumor’s anatomical site and burden. In recent years, the number of clinical trials investigating this delivery method for solid tumors has grown substantially [43]. An additional advantage of locoregional delivery is the potential to reduce or eliminate the need for lymphodepleting chemotherapy, since robust systemic expansion is less critical in this context. In a Phase I trial, Adusumilli et al. evaluated intrapleural administration of mesothelin-targeted CAR-T cells in patients with malignant pleural disease, combined with the anti-PD-1 antibody pembrolizumab [44]. The treatment was well tolerated, with no severe CRS or dose-limiting toxicities observed. CAR-T cells demonstrated local expansion, systemic trafficking, and antitumor activity, including durable partial responses and disease stabilization in heavily pretreated patients. In another clinical study, Brown et al. investigated locoregional delivery of IL-13Rα2-targeted CAR-T cells in patients with recurrent high-grade glioma [45]. In this Phase I trial, CAR-T cells were infused directly into the tumor cavity or ventricular system, achieving localized exposure while minimizing systemic toxicity. The treatment was well tolerated, with no severe neurotoxicity or CRS, and several patients experienced radiographic tumor regression and prolonged survival compared with standard outcomes. In pediatric and young-adult patients with diffuse intrinsic pontine glioma, Vitanza et al. administered ex vivo gene-modified autologous T cells expressing a CAR against B7-H3 (CD276) via repeated intracerebroventricular infusions, demonstrating durable feasibility, manageable toxicity, and local immune activation with clinical benefit [46]. Despite these encouraging results, conclusions are constrained by the small, single-center Phase 1 cohort without a control arm and variation in treatment timing (pre- vs. post-progression), which complicate the attribution of clinical benefit and limit broader applicability. Although locoregional administration of CAR-T cells can enhance on-target exposure at the tumor site, improve trafficking across anatomic barriers, and reduce systemic cytokine release and off-tumor toxicity, its therapeutic impact is often limited by poor control of micrometastatic disease, and the persistence of immunosuppressive local microenvironments. In addition, technical and anatomical constraints, such as catheter placement, obstruction, or infection risk, may restrict repeat dosing and broad applicability. Consequently, locoregional CAR-T therapy is unlikely to be curative as a stand-alone approach for most cancers and should instead be deployed within integrated strategies, for example, in combination with low-dose systemic CAR-T cells, immunomodulatory agents, or regional conditioning (e.g., low-dose intratumoral chemotherapy), to achieve more durable and comprehensive tumor control.

## 4. Targeted In Vivo CAR Delivery to T Cells

Recent advances have enabled in vivo gene therapies capable of directly reprogramming T cells into CAR-T cells within the patient [48]. This approach obviates the need for lymphodepletion and mitigates the toxicities associated with chemotherapy-based preconditioning, thereby addressing key limitations of conventional ex vivo manufacturing. In general, in vivo CAR-T generation uses off-the-shelf reprogramming cassettes, making it substantially faster by several weeks than current ex vivo CAR-T manufacturing processes (Figure 2B). The platform can incorporate a range of delivery modalities, including both viral and non-viral vectors, each with its own advantages and limitations [49]. Adeno-associated viruses (AAVs) are widely used for in vivo gene delivery because they can support long-term transgene expression primarily by persisting as episomal DNA, typically without integrating into the host genome, in both dividing and non-dividing cells [50]. Lentiviral vectors are also a leading gene-therapy platform, largely due to their capacity to stably integrate relatively large genetic cargos into the host-cell genome. In addition, they offer broad tropism and high transduction efficiency in both dividing and non-dividing cells [51]. Cell targeting in vivo can be achieved by displaying high-affinity antibodies on the surface of vector particles, enabling recognition of specific receptors expressed on the desired cell population (e.g., T cells, NK cells). As proof of concept, Pfeiffer et al. demonstrated that a CD8-targeted, CD19-CAR-encoding lentiviral vector could transduce T cells in vivo in mice and mediate effective B cell depletion [52]. Agarwalla et al. developed an implantable biomaterial scaffold, termed MASTER, designed to generate CAR-T cells in vivo by providing a supportive microenvironment for T cell activation, viral transduction, proliferation, and release into circulation. In preclinical models, CAR-T cells produced from the scaffold persisted in the bloodstream and mediated tumor control in distal lymphoma xenografts, demonstrating functional activity comparable or superior to conventional ex vivo gene-manufactured CAR-T cells [53,54].

Nanosystems such as lipid nanoparticles (LNPs) offer a safer non-viral in vivo gene delivery platform for T cell engineering, overcoming many of the limitations associated with viral-based methods [55]. Like viral vectors, delivery specificity can be enhanced by conjugating LNPs with ligands, such as monoclonal antibodies, scFvs, or nanobodies, that target T cell markers including CD3, CD8, CD5, CD7, and PECAM-1 [56,57,58,59]. CD7 targeting enables delivery to both T cells and NK cells. One prominent platform, NCtx, co-encapsulates minicircle DNA encoding a CAR together with SB100x transposase mRNA in dual-targeted LNPs, achieving durable CAR integration and potent anti-leukemic activity in humanized mouse models following a single intravenous dose [60]. Billingsley et al. developed LNPs functionalized with anti-CD3 antibody fragments to selectively target T cells in vivo, enabling efficient delivery of CAR mRNA and inducing transient yet therapeutically meaningful CAR expression [61]. In mouse models, CD3-targeted LNPs depleted B cells by up to 90% and produced strong antitumor responses. Hunter et al. demonstrated the feasibility of in vivo CAR-T cell generation using targeted LNPs carrying mRNA encoding an anti-CD19 CAR (CPTX2309) [62]. The LNPs were designed to selectively bind CD8^+^ T cells. In mouse and non-human primate models, a single intravenous dose induced rapid CAR expression in circulating CD8^+^ T cells, leading to profound and durable B cell depletion followed by the reconstitution of naïve B cells, with minimal cytokine release or systemic toxicity. Similarly, Chen et al. developed a non-viral strategy to generate humanized CD19 CAR-T cells using mRNA-laden LNPs. The engineered CAR-T cells showed strong cytotoxicity against CD19-positive leukemic cells in vitro and effectively eliminated tumors and prolonged survival in mouse models [63]. Many systemically delivered lipid nanoparticles tend to accumulate in the liver, which may increase the risk of liver toxicity depending on formulation and dose. Interestingly, Hamilton et al. demonstrated that antibody-targeted Cas9-EDVs (Engineered Enveloped Delivery Vehicles) can generate genome-edited CAR-T cells in humanized mice with no off-target delivery to liver hepatocytes [48]. These Cas9-EDVs are virally derived, antibody-targeted particles that package pre-formed Cas9–gRNA ribonucleoprotein complexes along with CAR-encoding genes. Although these advances were made in mice, they show how gene-therapy tools can directly reprogram immune cells in vivo into CAR-expressing cells using non-viral vectors.

Building on these preclinical studies, Xu et al. conducted a first-in-human Phase 1 trial evaluating ESO-T01 product, a lentiviral vector designed to enable in vivo generation of BCMA-targeted CAR-T cells, in patients with R/R multiple myeloma [64]. Among four patients treated at the lowest dose without lymphodepletion, all responded with two achieved complete responses and two partial responses. Treatment-related CRS occurred in all patients, along with mild neurotoxicity, but no grade 4 or fatal events. Similarly, JY231, another in vivo CAR-T lentiviral construct delivering an anti-CD19 CAR for B cell malignancies, showed early promise: in a patient with R/R diffuse large B cell lymphoma, CAR-T generation was confirmed, and complete remission occurred by day 35 post-treatment [65]. Likewise, the mRNA-LNP-based in vivo CAR-T therapy HN2301 showed promising results in a small cohort of five patients with refractory SLE. CD19-CAR mRNA delivered via targeted LNPs reprogrammed up to ≈60% of CD8^+^ T cells into CAR-T cells within six hours [66]. This led to near-complete depletion of circulating B cells for 7–10 days, and substantial reductions in blood autoantibody levels (such as anti-dsDNA). No grade ≥ 3 CRS, neurotoxicity, or other severe adverse events were observed. An ongoing Phase 1, open-label, dose-escalation trial is evaluating intravenous administration of LNP-formulated mRNA encoding a glypican-3 (GPC3)-specific CAR (MT-303) in adults with advanced or metastatic GPC3-expressing tumors (NCT0647869) [67]. Another Phase 1 clinical study is also underway to evaluate the safety, tolerability, and pharmacologic activity of CPTX2309 in healthy volunteers (NCT06917742). This trial was built on strong preclinical evidence showing that CPTX2309’s CD8-targeted LNP platform can efficiently deliver mRNA encoding to an anti-CD19 CAR, generating functional CAR-T cells in vivo as discussed above [62].

Most in vivo CAR-T studies are still limited to preclinical models, and long-term safety, biodistribution, and persistence of transduced cells remain incompletely characterized. Achieving selective delivery of CAR transgenes to T cells or NK cells is a major challenge, as off-target transduction of immunosuppressive populations, such as regulatory T cells (Tregs) or myeloid-derived suppressor cells, could blunt antitumor activity. Similarly, accidental transduction of malignant cells can lead to immune escape, as illustrated by a pediatric B-ALL patient in whom a single leukemic B cell was transduced ex vivo with an anti-CD19 CAR, resulting in “cis” masking of CD19, clonal expansion, and relapse with functional resistance [68]. Viral delivery strategies also carry specific safety concerns, including insertional mutagenesis and immune responses against the vector that could reduce CAR expression. From a dosing perspective, a single administration of a lentiviral vector may provide durable CAR expression, whereas repeated dosing, particularly with nanocarriers, may increase anti-carrier or anti-CAR immunity, which may reduce effectiveness or tolerability overtime. Incorporating safety switches, such as inducible caspase-9 (iCasp9) or herpes simplex virus thymidine kinase, enables the selective elimination of transduced cells in the event of severe toxicity or malignant transformation. Despite these challenges, several lentiviral CAR constructs targeting defined immune cell populations are currently under clinical ivestigation, demonstrating the potentiel of in vivo CAR-T approaches [54]. These early studies are expected to inform critical refinements in vector design, targeting specificity, and safety controls, thereby guiding the development of more effective and safer therapies. Table 1 compares ex vivo and in vivo CAR-T platforms.

## 5. mRNA CAR-T Engineering

As noted above, mRNA-engineered CAR-T cells are emerging as a promising alternative to viral- or DNA-based platforms, offering distinct advantages in terms of safety and flexibility. Unlike integrating vectors, mRNA enables efficient but transient CAR expression without genomic insertion, thereby avoiding the risk of insertional mutagenesis and other long-term toxicities. This short-lived expression window, typically lasting only a few days, allows for iterative dosing and fine-tuning of therapeutic activity, reducing the incidence of prolonged toxicity and T cell exhaustion [69]. From a manufacturing perspective, mRNA CAR-T production is faster, more scalable, and more cost-efficient than viral vector-based methods, benefiting from the technological advances and infrastructure established during the rapid development of COVID-19 mRNA vaccines [70]. The modular nature of mRNA constructs enables rapid modification of CAR designs, including the incorporation of multiple CAR constructs, safety switches, and auxiliary molecules such as cytokines or chemokine receptors. This flexibility accelerates preclinical optimization and supports the development of more personalized therapies. Optimization of mRNA sequences, combined with the incorporation of modified nucleosides such as pseudouridine (ψ) and 1-methyl-ψ, has further enhanced CAR expression and achieved near-complete antitumor efficacy in animal models, underscoring the promise of mRNA therapies for in vivo CAR-T cell generation [71]. These features position mRNA-based CAR-T platforms as a versatile foundation for next-generation cellular immunotherapies.

With respect to delivery, electroporation has been widely employed to introduce mRNA or DNA into T cells, enabling efficient yet transient CAR expression and enhancing safety by avoiding permanent genomic integration [72,73]. However, limitations such as electroporation-induced cell death and its unfeasibility for in vivo use have prompted the development of alternative delivery strategies. In this respect, lipid-based platforms have emerged as promising non-viral approaches for both ex vivo and in vivo delivery. Among these, ionizable LNPs represent a major advancement over conventional cationic or neutral LNPs owing to their pH-responsive charge properties and optimized lipid composition [74,75]. Their neutral charge at physiological pH minimizes nonspecific interactions and systemic toxicity, whereas protonation in the acidic endosomal environment promotes electrostatic interactions with the endosomal membrane, facilitating efficient cargo release into the cytosol. This improved endosomal escape yields substantially higher gene expression in vivo, positioning ionizable LNPs as highly effective vehicles for mRNA delivery and T cell engineering. As a result, most, if not all, emerging in vivo platforms employ ionizable LNPs.

In terms of clinical applications, two trials (NCT03448978 and NCT04146051) have evaluated Descartes-08, an anti-BCMA CAR-T cell therapy developed by Cartesian Therapeutics that employs mRNA-based engineering via electroporation [76]. In the NCT03448978 study, patients with R/R multiple myeloma received Descartes-08 infusions, with or without lymphodepletion, demonstrating a favorable safety profile and encouraging antitumor activity [77]. The subsequent NCT04146051 trial expanded the use of this platform to autoimmune disease, specifically generalized myasthenia gravis, where repeated dosing resulted in significant clinical improvement with minimal toxicity [78,79]. More recently, the HN2301 trial utilized a T cell-targeted ionizable LNP platform developed by MagicRNA to deliver CD19 CAR mRNA directly in vivo (NCT06801119). As indicated above, this approach enabled the successful generation of functional CAR-T cells within patients, underscoring the central role of ionizable LNPs in achieving safe and efficient systemic gene delivery [66]. Collectively, these trials showed that mRNA-engineered CAR-T cells can elicit potent yet controllable therapeutic effects, highlighting the potential of transient CAR expression to enhance safety and broaden the applicability of cell therapies beyond oncology. Nonetheless, interpretation of these findings is limited by the early-phase nature of the studies, including small patient cohorts, largely preliminary efficacy readouts, and relatively short follow-up for assessing durability and late toxicities. Additional challenges include heterogeneity in treatment regimens and the repeat-dosing requirements inherent to transient mRNA expression, which may affect feasibility and comparability across cohorts. There is also a need for precise cell-type targeting to avoid unintended transfection of non-target cells. Scalability remains a practical concern, as large-scale production of mRNA CAR-T products for repeated dosing requires robust and reproducible manufacturing processes. Although non-viral delivery methods are generally considered to carry no risk of insertional mutagenesis, repeated infusion may still trigger anti-vector immunogenicity, potentially reducing CAR expression or limiting re-dosing. The field would benefit from standardized assays to enable cross-platform comparison. Table 2 summarizes the key features of mRNA-based CAR-T cell therapy compared with DNA- or viral-based approaches.

## 6. Beyond CAR-T: CAR-NK and TCR-T as Alternative Engineered Cell Therapies

CAR-T cell therapies have achieved major clinical success, particularly in hematologic malignancies. Their broader use, however, is constrained by serious toxicities and by the requirement for tumor antigens that are accessible on the cell surface. These limitations have accelerated the development of alternative engineered immune cell platforms. One leading approach is CAR-NK cell therapy, which has “off-the-shelf” potential because NK cells can be generated as allogeneic products from healthy donors and multiple sources, including peripheral blood, umbilical cord blood, and induced pluripotent stem cells [80,81]. Early studies also suggest a safer profile. In a landmark CD19 CAR-NK study, patients showed clinical responses without CRS, neurotoxicity, or graft-versus-host disease (GvHD) [80]. Unlike allogeneic CAR-T products, allogeneic CAR-NK cells typically do not require TCR gene editing to reduce the risk of GvHD, which simplifies the manufacturing process. Furthermore, NK cells retain their natural cytotoxic functions alongside CAR-mediated recognition and can also mediate antibody-dependent cellular cytotoxicity via CD16, enabling combination strategies with monoclonal antibodies [82]. So far, early clinical experience shows good safety and early signs of benefit for CAR-NK therapies [83]. However, CAR-NK therapies also have limits. They often persist for a shorter time in the body. This can reduce durability and may require repeat dosing. Solid tumors remain challenging. NK cells may not traffic or infiltrate tumors well, and the TME can suppress their activity. These barriers may require added engineering or combination strategies.

Another major platform is TCR-T cell therapy, in which T cells are engineered to express tumor-specific T cell receptors (TCRs) [84]. Unlike CARs, TCRs recognize peptides presented by major histocompatibility complex (MHC) molecules, expanding the target landscape to include epitopes derived from both membrane and intracellular proteins. TCR-T cells also typically require lower epitope density for activation than CAR-T cells [85]. TCR affinity is often described as a paradox. Adequately strong binding is needed to sustain T cell proliferation and support tumor eradication, yet excessively high affinity can promote early T cell exhaustion after antigen engagement. Moreover, higher affinity increases the risk of off-target or cross-reactive recognition of self-antigens, potentially damaging healthy tissues. This concern -is particularly relevant for alloreactive TCRs, which were not tuned through thymic selection against self MHC–peptide complexes [86]. In contrast to CAR therapies, TCR-T cell therapy is limited by HLA restriction, meaning only patients with compatible HLA alleles are eligible for a given cell product. With respect to treatment failure, secondary or acquired resistance mechanisms represent a major concern. A dominant escape mechanism is loss or downregulation of MHC class I on tumor cells, which prevents presentation of the target epitope and thereby abrogates TCR-T recognition [84]. Safety also remains a major concern, as severe toxicities have been reported in engineered TCR programs. One case involved fatal cardiac toxicity linked to titin cross-reactivity in an affinity-enhanced TCR approach [87]. Another report described a fatal serious adverse event after infusion of MART-1-specific TCR T cells [88]. Moreover, introduced TCR chains can mispair with endogenous TCR chains, generating unintended specificities and increasing the risk of off-target reactivity. Allogeneic TCR-T cells may induce GvHD. Accordingly, additional engineering strategies, such as gene editing to eliminate native TCR expression or enforce correct chain pairing are required to mitigate these risks [89]. Table 3 presents a comparative analysis of CAR-T cells, CAR-NK cells, and TCR-T cells.

## 7. Challenges and Emerging Solutions

Efforts to apply CAR-T cell therapy to solid tumors have yielded disappointing clinical outcomes and uncovered several major challenges that limit its therapeutic efficacy. These include poor trafficking and infiltration of CAR-T cells into tumor sites, immunosuppression mediated by the tumor microenvironment, as well as tumor antigen heterogeneity and loss. Such limitations affect both DNA- and mRNA-engineered CAR-T cell platforms. In the context of autoimmune diseases, CAR-T cell therapy faces a distinct yet equally complex set of challenges. These include achieving precise antigen specificity without inadvertently depleting protective immune subsets, maintaining an optimal balance between persistence and controllability of CAR-T cell function, and mitigating the risk of chronic inflammation or secondary immunodeficiency. Beyond these biological and mechanistic hurdles, practical and translational considerations, including complicated manufacturing processes, high production costs, and interpatient variability in T cell quality and expansion, further constrain scalability and accessibility. Moreover, the use of certain DNA technologies (e.g., viral vectors, CRISPR) in CAR-T cell engineering raises important ethical concerns. These include the potential for off-target mutations, challenges in ensuring informed consent for therapies with uncertain long-term effects, and inequities in access due to high costs. The following subsections outline technological innovations aimed at overcoming these challenges.

### 7.1. Limited Tumor Infiltration and Retention

While hematologic malignancies offer relatively accessible targets, solid tumors present substantial physical and biochemical barriers that limit CAR-T cell efficacy. One major obstacle is inefficient CAR-T cell infiltration/migration into tumor sites, largely due to mismatched chemokine receptor expression that prevents T cells from responding effectively to tumor-derived chemotactic signals [90]. In addition, the dense extracellular matrix (ECM) and surrounding stroma form a physical barrier that further restricts T cell infiltration [91,92].

The ECM in solid tumors consists of a complex, tightly packed network of fibrous proteins, such as collagens and laminins, along with proteoglycans and glycosaminoglycans [92,93]. To overcome this, one promising strategy is to engineer CAR-T cells to express enzymes that degrade specific ECM components, thereby facilitating their penetration into tumor tissue. A notable example involves the development of heparanase-expressing CAR-T cells, in which T cells are modified to co-express human heparanase (HPSE) with the CAR construct [94]. Heparanase degrades heparan sulfate proteoglycans, enabling T cells to migrate more effectively through the ECM. In preclinical models of neuroblastoma and melanoma, HPSE-engineered CAR-T cells exhibited enhanced tumor infiltration and superior antitumor activity compared to conventional CAR-T cells, without causing significant systemic toxicity. Similar approaches have employed other ECM-degrading enzymes. For instance, CAR-T cells engineered to express hyaluronidase (PH20) improved infiltration into pancreatic and breast tumor models by breaking down hyaluronic acid within the tumor stroma [95]. Van Pelt et al. recently showed that the co-expression of matrix metalloproteinase-7 and osteopontin can enhance CAR-T cell penetration into solid tumor models by promoting ECM remodeling [96]. These modifications enhanced T cell extravasation, interstitial migration, and tumor infiltration, resulting in improved tumor control and prolonged survival in a neuroblastoma model. The engineered CAR-T cells target GD2, a disialoganglioside highly expressed in neuroblastoma and several other tumors, but with limited expression in normal tissues [97]. Additionally, targeting fibroblast activation protein (FAP)-expressing cancer-associated fibroblasts (CAFs) has been shown to remodel the tumor stroma and promote T cell infiltration in tumor models [98]. While ECM modulation is a promising strategy to enhance CAR cell therapy in solid tumors, it should be approached carefully, as ECM-targeting enzymes can produce unpredictable effects. Clinical trial outcomes have been variable, highlighting the need for additional studies to better establish efficacy and more clearly characterize potential toxicities of these approaches [99].

Beyond extracellular matrix (ECM) remodeling, CAR-T trafficking can be improved by engineering cells to overexpress chemokines, chemokine receptors, or adhesion molecules, and these approaches have shown promising preclinical results. For example, GD2-targeted CAR-T cells engineered to express the CCR2b receptor exhibited more than a ten-fold increase in migration toward CCL2-secreting neuroblastoma, which was associated with greater intratumoral accumulation and enhanced antitumor efficacy [100]. Likewise, CXCR1 or CXCR2 expression enabled CAR-T cells to follow tumor-derived IL-8 signals, improving migration and tumor control [101]. In a related approach, co-expression of IL-7 with CCL19 or CCL21 improved CAR-T cell survival and intratumoral presence, leading to stronger antitumor activity [102,103]. Overall, enhancing CAR-T cell infiltration through chemokine/cytokine receptor engineering or cytokine/chemokine secretion (armored CARs) remains highly promising but largely preclinical. Although early Phase I/II trials are emerging, the clinical evidence is still limited and heterogeneous, and no approach has become standard of care [104,105,106,107]. Moreover, even when trafficking improves, major hurdles persist, including antigen heterogeneity and off-tumor toxicity, and an immunosuppressive TME. Locoregional administration provides an additional means to bypass trafficking barriers (see Section Local Administration of CAR-T Cells). For instance, in glioblastoma, local CAR-T delivery improved therapeutic activity compared with intravenous infusion in a clinical study [108]. Of note, unlike CAR-T cells, CAR-macrophages (CAR-Ms) can efficiently infiltrate solid tumors and remain active within the tumor microenvironment (TME) [109]. However, unlike CAR-T cells, which can proliferate and persist long-term, CAR-Ms are terminally differentiated, with limited lifespan and expansion capacity, which may constrain their broad use.

### 7.2. Immunosuppressive TME

Even when CAR-T cells successfully infiltrate solid tumors, they encounter an immunosuppressive TME. Tumors secrete inhibitory cytokines, recruit regulatory immune cell populations, and express checkpoint ligands, all of which impair CAR-T cell function. To overcome these barriers, several engineering strategies have been developed. Among them, “armoring” CAR-T cells to secrete therapeutic payloads or to express synthetic receptors that integrate additional signals is a straightforward approach to target the immunosuppressive TME. In preclinical solid tumor models, CAR-T cells secreting IL-12 have showed enhanced proliferation, persistence, cytotoxicity, and survival, as well as resistance to PD-L1-mediated inhibition [110]. Similarly, CAR-T cells engineered to secrete IL-18 exhibited enhanced in vivo expansion and persistence, along with improved recruitment of endogenous antitumor immune effectors, thereby reshaping the TME to promote a more potent and broad immune response [111].

Interleukin-15 (IL-15) is a cytokine essential for the development, survival, and activation of T cells and natural killer (NK) cells [112]. It promotes the proliferation and maintenance of memory CD8^+^ T cells, enhances NK cell cytotoxicity and homeostasis, and supports the generation of memory-like CAR-T cells that may improve long-term persistence and reduce relapse risk. Given these functions, IL15-armored CAR-T cells are engineered to secrete IL-15, thereby improving the survival, proliferation, and effector activity of T cells and NK cells, particularly within the immunosuppressive TME [113]. In preclinical models of lung cancer, neuroblastoma, and glioblastoma, IL15-secreting GD2-CAR-T cells demonstrated increased accumulation within the TME and exhibited enhanced cytotoxic activity [114,115]. Similarly, vascular endothelial growth factor receptor 2 -targeted CAR-T cells expressing IL-15 displayed markedly improved survival, proliferation, persistence, and antitumor efficacy compared with conventional CAR-T cells [116]. In a recent Phase I trial, IL-15-armored GPC3-CAR-T cells outperformed conventional CAR-T cells by resisting the immunosuppressive TME, expanding more robustly, persisting longer, and accumulating in a functional, metabolically fit state, leading to tumor regressions and disease control in a subset of patients [117]. Hence, these results position IL-15 armoring as a promising strategy to overcome TME-mediated suppression in solid tumor CAR-T therapy.

Using CRISPR-mediated knock-in, Chen et al. recently introduced a novel strategy to overcome immunosuppression by harnessing endogenous tumor-restricted promoters (such as NR4A2 and RGS16) to drive the localized expression of proinflammatory cytokines (IL-12 and IL-2) exclusively upon CAR-T cell activation within the TME [118]. This approach enhanced CAR-T cell polyfunctionality and infiltration promoted epitope spreading to stimulate host antitumor immunity. Although preclinical, the method was shown to be feasible in patient-derived T cells, underscoring its translational potential. In parallel, combining CAR-T cell therapy with immune checkpoint inhibitors, such as anti-PD-1 agents (e.g., pembrolizumab), has emerged as another promising approach to counteract TME-mediated suppression. Early clinical trials in solid tumors have demonstrated that such combinations can reduce T cell exhaustion and sustain cytotoxic function [119,120]. Building upon this concept, CAR-T cells engineered to secrete PD-1-blocking scFvs within the TME enhanced the survival of PD-L1-tumor-bearing mice in syngeneic and xenogeneic mouse models [121]. This strategy may also improve safety, as the locally secreted scFv restricts PD-1 blockade to the tumor site, potentially avoiding toxicities associated with systemic immune checkpoint inhibition. CAR-T cells have also been genetically modified to delete or reprogram inhibitory receptors such as PD-1 or LAG-3, further enhancing their resistance to TME-induced dysfunction [122,123]. Additionally, CAR-T cells engineered with decoy receptors that bind and neutralize immunosuppressive factors, such as transforming growth factor-β (TGF-β), have demonstrated increased proliferation, enhanced cytokine secretion, resistance to exhaustion, prolonged persistence, and potent tumor eradication in aggressive human prostate cancer models [124].

Among the immunosuppressive cells recruited to tumors, tumor-associated macrophages (TAMs), particularly the M2 subset, represent the most abundant immune-infiltrating cells within the TME [125,126]. M2 macrophages suppress immune responses by secreting cytokines such as IL-10 and TGF-β, producing enzymes like arginase 1 and indoleamine 2,3-dioxygenase, recruiting Tregs, and supporting the survival of myeloid suppressor cells [125]. All together contributing to the formation of an immunosuppressive niche that limits the efficacy of CAR-T cells. Hence, targeting M2 macrophages with CAR-T cells has emerged as a promising strategy to enhance antitumor immunity. In this respect, recent studies have developed CAR-T cells targeting macrophage-specific markers, such as F4/80 and CD147, to selectively deplete M2 macrophages. F4/80-targeted CAR-T cells effectively eliminated TAMs in mouse models, delaying tumor growth and extending survival [127]. Similarly, PD-L1-targeted CAR-T cells have been engineered to attack both tumor cells and M2 macrophages, achieving robust tumor eradication across multiple solid tumor models [128].

Like M2 macrophages, CAFs suppress CAR-T cell activity by releasing immunosuppressive cytokines (e.g., IL-10, TGF-β), secreting tumor-promoting growth factors, and depositing extracellular matrix components that hinder immune cell infiltration and facilitate metastasis [129]. Huang et al. recently demonstrated that a universal anti-fluorescein CAR-T cell platform, when combined with two bispecific adapters, one targeting tumor cells via folate/FRα and the other targeting CAFs via a FAP ligand, can simultaneously eliminate cancer cells and CAFs [130]. This dual-targeting approach enhanced tumor regression, improved CAR-T cell infiltration, proliferation, and activation, and achieved superior efficacy in both “hot” and “cold” solid tumor models without significant toxicity. Notably, targeting CAFs, such as through FAP, has been proposed to enhance immunotherapy efficacy [131]; however, developing separate CAR-T cells, one against CAFs and the other against tumor cells, can be complex and costly.

Collectively, these complementary strategies, ranging from transcriptional control of cytokine release to checkpoint modulation and cytokine neutralization, as well as approaches that deplete or reprogram suppressive stromal and myeloid compartments, are supported primarily by strong preclinical evidence. Emerging clinical evidence, including a Phase I trial of IL-15-armored GPC3-CAR-T cells and early -studies combining CAR-T therapy with the PD-1 blockade, demonstrates the feasibility of TME-directed approaches and suggests meaningful antitumor activity in a subset of patients [117,120]. Moving forward, progress in solid tumor CAR-T therapy will likely depend on abondoning single-mechanism solutions in favor of integrated, safety- focused designs that enhance intratumoral T cell persistence and functionality while l mitigating systemic inflammatory toxicity. Such designs may incorporate tumor-restricted cytokine expression and safety switches for rapid CAR-T cell elimination if needed.

### 7.3. Antigen Heterogeneity, Tumor Escape, and On-Target, Off-Tumor Toxicity

Antigen heterogeneity and on-target, off-tumor toxicity represent two major barriers to the long-term efficacy and safety of CAR-T cell therapy. Tumors frequently display variable antigen expression across malignant cells, leading to incomplete targeting and disease relapses. In addition, tumor cells may downregulate or delete the primary target antigen, thereby escaping immune recognition. This phenomenon is well documented in current CAR therapies: CD19 CAR-T cells show relapse rates of 40–60%, often associated with antigen loss or limited CAR-T cell persistence [132]. Similarly, BCMA-directed CAR-T-therapies are similarly affected by antigen loss and the immunosuppressive TME [133,134]. At the same time, most tumor-associated antigens are also expressed at low levels in normal tissues, creating a risk of on-target, off-tumor toxicity; for example, CD19 CAR-T cells deplete both malignant and healthy B cells, causing B cell aplasia that necessitates long-term immunoglobulin replacement therapy [135]. This challenge is even more pronounced in solid tumors, where supportive care options are limited.

**Figure 3:**
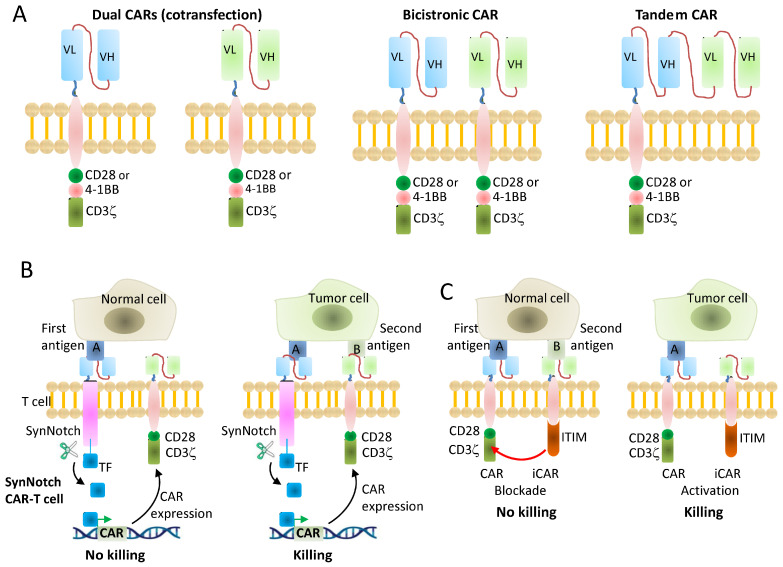
(**A**–**C**). (**A**) Illustration of dual-targeting of CAR-T cell strategies. Several strategies have been described including two CAR-T cell products individually transduced with two different vectors (cotransfection), one bispecific CAR-T cell product expressing one bivalent CAR with two scFvs targeting two different tumor antigens produced by transduction of a bicistronic vectors to introduce two separate CARs with one antigen-binding domain per CAR, or by fusing the VL-VH of one single scFv directly to the VL-VH of the other scFv defined as bivalent tandem. (**B**) The SynNotch–CAR-T mechanism works as a two-step “AND-gate.” First, the engineered SynNotch receptor binds a priming antigen A, leading to the release of the intracellular transcription factor (TF) from the receptor’s cytoplasmic domain. Then the TF enters the nucleus and turns on CAR expression. Next, the expressed CAR is transported to the T cell membrane, where it can bind a second antigen B on the tumor cell. When the CAR engages this second antigen, the T cell becomes fully activated and releases perforin and granzymes to kill the tumor cell. This design improves targeting specificity by ensuring CAR expression, and therefore robust activation, occurs only after SynNotch engagement. (**C**) Inhibitory CARs (iCARs) that incorporate ITIM signaling motifs are engineered to recognize antigens expressed on healthy tissues but absent from tumor cells. When the iCAR binds its cognate normal-tissue target via the second antigen B, it delivers an inhibitory signal that suppresses signaling from a co-expressed CAR in the same T cell. Because the iCAR antigen B is restricted to normal cells, inhibition is engaged in healthy tissues, while CAR-T activation is preserved in tumors.

To mitigate both antigen escape and on-target, off-tumor toxicity, several engineering strategies have been developed. Multi-antigen targeting can be achieved by administering CAR-T cells directed against a second antigen [136] either by dual CAR-T cells co-expressing two complete CAR molecules [137] or by tandem CARs that combine two scFvs within a single receptor (Figure 3A) [138]. These designs effectively implement OR logic, enabling CAR-T cells to recognize either (or both) antigens, thereby improving coverage of heterogeneous tumors and reducing the likelihood of immune escape due to antigen loss, which has been observed after single-antigen CAR therapy in B-ALL, non-hodgkin lymphoma (NHL), and multiple myeloma [133,139,140].

AND-gated CAR-T cells can further enhance specificity by triggering robust activation only when two (or more) antigens are co-expressed on tumor cells, ideally with minimal overlap on normal tissues [141,142,143,144,145]. Optimizing split-signaling (AND-gated) dual CAR architectures, in which antigen recognition and costimulatory signaling are partitioned across distinct receptors, has been shown to improve specificity, antitumor efficacy, and safety [146]. Despite progress in logic-gated CAR-T design, challenges such as construct complexity and the difficulty of tuning signaling strength and activation thresholds have limited clinical translation. The synthetic notch (SynNotch) receptor, used in combination with CARs, can provide an additional layer of specificity by coupling antigen sensing to conditional CAR expression, enabling T cells to respond dynamically to their environment [147] (Figure 3B). Several SynNotch–CAR constructs have shown promising preclinical results but still require clinical validation [148]. Of note, for AND and OR gates, including SynNotch systems, antigen selection is crucial because inadequate or uneven expression can compromise gate function.

An alternative strategy to control tumor specificity involves incorporating an inhibitory “off-switch” that suppresses CAR signaling in normal tissues expressing a protective antigen, while permitting activation in tumor tissues lacking this antigen, consistent with NOT-gate logic. This principle is analogous to the mechanism by which NK cells avoid attacking healthy “self” tissues via inhibitory receptors such as KIRs [149]. A representative example is the use of inhibitory CARs (iCARs) that incorporate inhibitory intracellular signaling motifs (ITIM-containing domains) from checkpoint or inhibitory receptors (PD-1, CTLA-4, TIGIT, BTLA, LIR-1) to actively dampen cytotoxicity in healthy tissues [150,151,152,153,154,155] (Figure 3C). Careful antigen selection is essential, since inadequate or uneven expression may impair inhibitory function. In parallel, some approaches restrict activity through conditional activation rather than antigen-logic gating. Inducible Casp9 functions as a pharmacologically controlled safety (suicide) switch, rather than a logic gate, enabling rapid elimination of CAR-T cells if severe toxicity occurs [156,157]. Masked CARs, in which the antigen-binding site is sterically shielded until unmasked by tumor-associated proteases [158], implement a NOT-like microenvironment-restricted activation strategy by limiting CAR engagement to protease-rich tumor sites.

As discussed above, current engineering strategies aim to (i) broaden coverage to limit escape (e.g., multi-antigen OR targeting) and (ii) tighten specificity to protect healthy tissues (e.g., AND/NOT gating, conditional activation, inhibitory circuits, and safety switches). For broad clinical adoption, these approaches must demonstrate reproducible patient-level benefit, scalable manufacturing, durable in vivo potency, and meaningful toxicity reduction without introducing new failure modes such as reduced sensitivity, double-negative escape, or unexpected cross-reactivity. Although complex CAR designs incorporating multiple regulatory mechanisms or vectors may address important safety and specificity challenges, increased architectural complexity often compromises robustness, scalability, and regulatory tractability, highlighting the need for careful cost–benefit evaluation in construct design

### 7.4. Autoantigen Specificity in Autoimmunity

In autoimmune diseases, the primary challenge is to eliminate pathogenic immune cells without broadly depleting normal populations. Achieving this specificity is complicated because many autoantigens are shared between healthy and pathogenic cells. One promising solution is CAR- Tregs which suppress autoimmune responses without causing global immunosuppression [159]. CAR-Tregs are engineered to express CARs that recognize specific autoantigens, enabling them to selectively suppress autoreactive T cells and maintain immune tolerance [160]. This targeted approach allows for the modulation of the immune response without broadly suppressing the entire immune system. Alternatively, CARs can be engineered to selectively target autoreactive B or T cells expressing disease-specific markers, preserving immune function while reducing autoimmune pathology [161]. In diseases like SLE, CD19 CAR-T cell therapy has shown promise in depleting autoreactive B cells, resulting in clinical improvement [12,13]. However, their limited antigen specificity also leads to the elimination of normal B cells. Hence, CAR affinity must be carefully calibrated, high enough to target pathogenic B cells effectively, yet low enough to avoid prolonged normal B cell aplasia. This principle also applies to B cell malignancies.

Idiotype-directed CAR-T therapy represents a precision immunotherapeutic strategy that exploits the unique antigenic determinants (idiotypes) of pathogenic B cell receptors. Each B cell clone expresses a surface immunoglobulin, typically IgM, with a distinct variable region defining its idiotype [162]. By generating anti-idiotype antibodies that specifically recognize this unique variable region, CARs can be engineered to selectively target and eliminate disease-associated B cell clones. In autoimmune diseases such as SLE, pathogenic B cells often utilize the VH4-34 heavy-chain variable gene segment. The monoclonal antibody 9G4, an anti-idiotypic antibody, specifically recognizes the idiotype encoded by VH4-34, thereby serving as a marker of autoreactive and pathogenic B cells.

**Figure 4:**
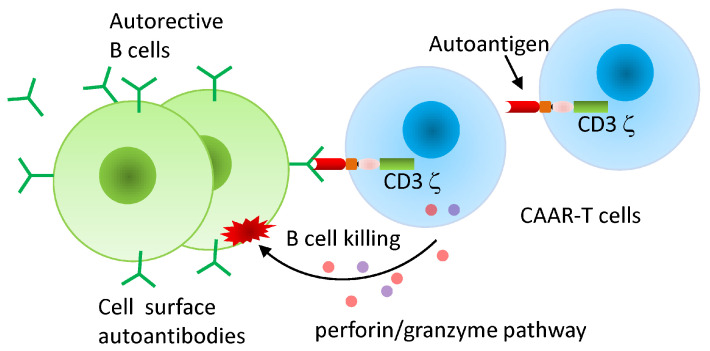
CAAR-T cells: Schematic representation of CAAR T cell specifically targeting autoreactive B cells. Autoreactive B cells often display their self-reactive IgM on their surface as B cell receptors (BCRs), which can then bind to the autoantigen and trigger the activation of CAAR-T cells and killing of B cells mainly via the perforin/granzyme pathway.

A recent proof-of-concept study by Liu et al. demonstrated that anti-9G4 CAR-T cells, derived from this anti-idiotypic antibody, selectively depleted autoreactive VH4-34-expressing B cells while sparing non-pathogenic populations [163]. These studies were conducted in preclinical models, including in vitro assays and humanized mouse systems, and provide early evidence of precision targeting. Unlike conventional anti-CD19 or anti-CD20 therapies, idiotype-specific CAR-T cells preserve broader humoral immunity by avoiding depletion of the entire B cell compartment. Although the individualized nature of idiotype generation presents manufacturing challenges, this approach offers a highly specific and promising avenue for the treatment of clonal B cell malignancies and autoantibody-mediated diseases. Similarly, Ellebrecht et al. introduced an innovative approach to treating autoimmune diseases by developing chimeric autoantibody receptor (CAAR)-T cells engineered to selectively eliminate autoreactive B cells responsible for disease (Figure 4) [164]. In preclinical studies on pemphigus vulgaris, T cells were engineered to express the autoantigen desmoglein 3 (Dsg3) on their surface, enabling precise recognition and destruction of B cells producing anti-Dsg3 autoantibodies. Importantly, this study has entered the clinical setting, making the first human application in pemphigus vulgaris under Phase 1 trial (NCT04422912). Early results indicate that the therapy is well tolerated, detectable CAAR-T cell persistence and early signs of disease improvement in some patients, though robust efficacy data are still pending. Building on this platform, Oh et al. extended CAAR-T therapy to MuSK myasthenia gravis, designing MuSK-CAAR-T cells that selectively eliminated MuSK autoantibody-producing B cells in preclinical models [165]. Together, these studies demonstrate that CAAR-T cell therapy can be tailored to different autoimmune diseases by incorporating disease-specific autoantigens, marking a major step toward personalized, antigen-specific immunotherapy, while noting that systematic clinical evaluation remains very limited.

The identification autoantibody specificities, using techniques such as peptide phage display or high-throughput antigen arrays, should guide the rational design of CAAR-T cells based on corresponding autoantigenic peptide epitopes [166,167,168]. By displaying disease-relevant peptide epitopes on their surface, engineered T cells can selectively recognize and deplete autoreactive B cells that produce pathogenic antibodies, while minimizing collateral immune suppression. In cancer, identifying antibody or autoantibody targets against tumor-associated antigens or neoantigens [169] can facilitate the development of conventional CAR-T cells, in which antibody-derived binding domains from single B cell clones are engineered to recognize and eliminate tumor cells expressing those targets [170,171]. Thus, while insights into antibody specificity enable antigen-selective immune modulation in autoimmunity, they also provide the foundation for cytotoxic targeting in oncology.

### 7.5. Balancing Persistence and Durability

Recent advancements in CAR-T cell therapy have focused on improving the balance between persistence and durability, which remains a major challenge, especially in solid tumors. Several strategies have shown promise in enhancing CAR-T cell longevity and therapeutic efficacy. Metabolic reprogramming, such as promoting oxidative phosphorylation, can induce favorable epigenetic and phenotypic changes that improve persistence [172]. In vivo restimulation, using vaccine-like approaches, helps maintain CAR-T cell activity within the TME, leading to more durable responses [173]. The development of allogeneic CAR-T cells provides a scalable alternative to autologous therapies, with ongoing efforts to enhance their longevity [174]. Armored CAR-T cells, engineered to secrete cytokines or other supportive factors, can resist immunosuppressive TME and sustain antitumor activity, often exhibiting enhanced persistence in vivo [175]. Optimizing costimulatory domains would be critical for achieving robust CAR-T cell proliferation and persistence, which strongly correlates with remission [176]. CAR-T cells engineered with different costimulatory domains, CD28 for shorter persistence or 4-1BB for longer persistence, should enable tailored therapeutic outcomes. Non-integrating vectors or mRNA-based CAR delivery would provide transient expression, balancing persistence with safety. Of note, high-affinity CAR-T cells may exhibit reduced in vivo persistence due to activation-induced exhaustion and apoptosis, while also increasing the risk of off-target interactions with healthy tissues [176,177]. Therefore, careful optimization of CAR specificity and affinity is essential for effective design.

The formation of T cell memory following CAR-T cell therapy is a critical determinant of long-term persistence and durable antitumor responses. After antigen-driven expansion and subsequent contraction, a subset of CAR-T cells differentiates into memory phenotypes, including central memory and stem cell-like memory cells, which exhibit enhanced self-renewal and longevity [178]. These memory CAR-T cells can persist for months to years, providing sustained immune surveillance and the capacity for recall responses upon antigen re-encounter. Memory development depends on both the starting T cell phenotype and CAR design. Using less differentiated T cells as the starting material, along with CAR constructs optimized through costimulatory domain selection, can bias differentiation toward durable memory rather than short-lived effector states [47,179]. The cytokine milieu during ex vivo expansion and after infusion, particularly IL-7, IL-15, and IL-21, further promotes memory formation and persistence. The enrichment of a stem cell memory phenotype at the time of infusion should enhance in vivo persistence and long-term antitumor efficacy. These outcomes are also shaped by antigen exposure kinetics and the TME, which can modulate memory differentiation and survival [180]. Persistence and proliferative capacity of CAR-T cells may be strengthened using fourth-generation CARs engineered for autonomous IL-7 and/or IL-15 secretion. However, constitutive expression of cytokines such as IL-7 or IL-15 in CAR-T cells removes normal physiological control, leading to antigen-independent signaling. This can cause excessive CAR-T cell expansion, increased risk of CRS and neurotoxicity, and unpredictable effects on bystander immune cells. Chronic cytokine exposure may also drive aberrant differentiation or functional exhaustion rather than durable memory. Therefore, tight control over the level, timing, and location of cytokine expression, for example, using inducible or regulated systems, is critical to balance efficacy with safety.

### 7.6. Manufacturing Complexities and Variability

Beyond biological hurdles, CAR-T therapies face substantial manufacturing, logistical, and delivery challenges that limit their scalability, accessibility, and consistency. Autologous CAR-T therapies, which rely on patient-specific T cells, involve a complex, multi-step process that includes cell purification, ex vivo modification, expansion, and reinfusion. This process is labor-intensive, costly, and time-consuming, often taking several weeks from leukapheresis to infusion, which can be critical for patients with rapidly progressing disease. Moreover, interpatient variability in T cell quality, fitness, and phenotype can significantly affect therapeutic outcomes and manufacturing success rates [181]. These factors collectively contribute to production bottlenecks, high prices, and limited global availability.

Emerging approaches, such as the in vivo engineering of CAR-T cells described in Section 4, aim to overcome these limitations by generating functional CAR-T cells directly within the patient, bypassing the need for ex vivo manipulation. This innovative strategy could simplify production, shorten turnaround times, and reduce costs, ultimately making CAR-T-therapies more accessible and scalable. In parallel, allogeneic CAR-T cells, often referred to as “off-the-shelf” therapies, represent a promising solution to the logistical and economic barriers associated with autologous products. Instead of using each patient’s own T cells, allogeneic CAR-T cells employ donor-derived T cells that are extensively gene-edited to eliminate endogenous TCRs to reduce the risk of GvHD and to evade host immune rejection [173,182,183]. This approach enables rapid availability, standardized large-scale manufacturing, and reduced interpatient variability, addressing many key scalability challenges and supporting broader clinical adoption. Batch production allows for consistent quality control and cost efficiencies. However, several challenges persist. Despite editing to reduce GvHD, immune rejection remains a major limitation, as the recipient’s immune system can recognize and eliminate allogeneic cells, leading to limited persistence and expansion in vivo compared with autologous products. Achieving durable engraftment often requires additional complex immune evasion engineering, which increases manufacturing complexity and regulatory burden. Moreover, extensive gene editing and multiplex modifications can introduce technical challenges and potential safety risks, such as off-target effects and genomic instability, necessitating stringent quality control [184]. Other limitations include variability in donor T cell qualities, challenges in selecting optimal donors, and the potential for residual low-level alloreactivity despite editing. With continued progress, however, allogeneic and in vivo engineered CAR-T platforms could transform the field of cellular immunotherapy, expanding its reach to more patients worldwide and paving the way for truly universal, accessible, and scalable cancer treatments.

## 8. Futures Perspectives

Future perspectives for CAR-T cell therapy point toward strategies that enhance precision, adaptability, and accessibility, while addressing the biological and practical limitations observed in current clinical use. Multi-antigen and logic-gated CAR designs are being refined to reduce relapses caused by antigen loss or heterogeneity and to improve tumor specificity, with approaches such as dual-CARs, tandem-CARs, and SynNotch systems offering highly programmable control of activity. At the same time, armored CARs engineered to secrete cytokines, reprogram suppressive immune populations, or resist checkpoint inhibition, are being tested to overcome the immunosuppressive TME, one of the greatest barriers in solid tumor therapy. Among emerging technologies, mRNA-based CAR platforms stand out as particularly promising, as they enable rapid and cost-effective manufacturing, transient expression for improved safety, and the flexibility to re-dose or adapt therapy in real time. Alongside these biological advances, improvements in scalability and accessibility are critical. The development of allogeneic “off-the-shelf” CARs, supported by advances in gene-editing to minimize GvHD and host rejection, could revolutionize availability by eliminating the need for patient-specific manufacturing. Coupled with closed, automated production systems, such innovations are expected to lower costs, reduce variability, and accelerate delivery of therapy to patients worldwide. Beyond T cells, next-generation approaches such as CAR-Tregs for autoimmunity, CAR-NK cells with lower toxicity risks and GvHD, and CAR-macrophages capable of enhancing phagocytosis and antigen presentation, hold potential to expand therapeutic applications while broadening the spectrum of diseases addressed. CAAR-T cells offer a novel, antigen-specific approach to treating autoimmune diseases. In the future, CAAR-T therapy could achieve durable, antigen-targeted remission and redefine the treatment paradigm for autoimmunity without compromising the patient’s immune response to infections. Of note, each strategy has its own advantages and disadvantages and should be tailored to the patient’s needs and health conditions.

With respect to immunosuppressive cells within the TME, co-targeting both tumor cells and supportive cell populations such as M2 macrophages with CAR-T cells, or combining CAR-T therapy with complementary interventions, represents a promising therapeutic strategy. Such combinatorial approaches may enhance CAR-T cell persistence and cytotoxicity within the TME, thereby improving overall efficacy and reducing tumor recurrence in solid malignancies. We anticipate that the continued integration of synthetic biology, gene editing, novel delivery methods (e.g., ionizable LNPs), improved manufacturing processes, and rational therapy combinations will further enhance the efficacy and safety of CAR-T cell therapies for both solid tumors and autoimmune diseases, while simultaneously reducing costs and increasing accessibility. Additionally, data emerging from ongoing clinical trials will facilitate a more comprehensive understanding of the constraints of designed CAR therapies, thereby enabling the creation of more precise and informative readouts for functional genetic screens.

Many CAR-T products have been evaluated in heavily pretreated patients, where prior chemo-immunotherapy, prolonged antigen exposure, and disease-related immune dysregulation can reduce T cell “fitness” (e.g., lower yield, skewed subsets, exhaustion), potentially impacting manufacturing robustness and in vivo performance. A forward-looking strategy is pre-emptive leukapheresis, collecting and cryopreserving T cells earlier in the disease course (for example at first high-risk relapse or before intensive salvage), so that higher-quality starting material is available if CAR-T is needed later. However, this approach also raises practical and ethical considerations: many banked collections may never be used (cost, storage, chain-of-identity, and governance burdens), product specifications and regulatory requirements can evolve over time, and clinical deterioration may still preclude later infusion regardless of cell quality. Prospective pathway studies are therefore needed to define who benefits most, the optimal timing of collection, and whether early banking translates into meaningful improvements in manufacturing success, toxicity, durability of response, and overall cost-effectiveness compared with standard “just-in-time” collection.

## Figures and Tables

**Table 1:** Comparison of ex vivo and in in vivo CAR-T cell platforms.

Category	Ex Vivo CAR-T	In Vivo CAR-T
Process	T cells are collected from the patient, genetically modified and expanded in a facility, then re-infused	A targeted vector or nanoparticle is infused directly into the patient to program T cells inside the body
Manufacturing	Labor-intensive GMP cell manufacturing with multiple quality checks	Mainly vector manufacturing, no external cell handling
Timeline	Typically 3–4+ weeks from collection to infusion	Could work within days as T cells are modified in situ
Cost	Very high due to individualized cell processing	Lower because the main product is a standard vector
Product variability	Depends on the patient’s starting T cell quality and expansion potential	More consistent since the “product” is uniform across patients
Patient eligibility	Some patients cannot provide enough healthy T cells for manufacturing	May treat patients with low T cell counts or heavily pretreated immune systems
Control over engineering	High control over CAR expression, activation state, and cell composition	Less precise control, depends on vector targeting and in-body biology
Lymphodepletion requirement	Routine and usually required to support engraftment and expansion	Not always required, but many early platforms still include it, some aim to eliminate need
Persistence	Can persist long-term in some patients, but persistence varies by manufacturing conditions, phenotype, and patient biology	Persistence depends on in vivo transduction/transfection efficiency, may achieve more physiological expansion and potentially longer persistence, but data are still early
Safety	QC improves reliability, but large doses of activated CAR-T cells can cause strong CRS and neurotoxicity	Avoids large cell infusions, main concerns are off-target gene delivery or uncontrolled in vivo expansion
Scalability	Difficult, each batch is patient-specific (autologous)	Highly scalable, vector can be manufactured in large batches
Repeat dosing	Rare due to cost, manufacturing burden, and immune rejection	Potentially easier to repeat vector administration
Clinical maturity	Multiple approved therapies, widely used	Early clinical testing, no approved products yet

**Table 2:** Comparison of CAR-T cell platforms.

Aspect	mRNA-Based CAR-T	DNA/Viral CAR-T
Genomic integration risk	None, mRNA remains cytosolic	Integrating viral vectors carry residual oncogenesis risk
Expression duration	Transient (days), allows dosing control but requires repeat administration	Persistence enables long-term effect but may lead to chronic toxicity
Nucleoside modifications	Yes, high translation efficiency	No
Safety profile	Enhanced, less toxicity (CRS, neurotoxicity), reduced T cell exhaustion, tunable levels	Higher risk due to prolonged CAR activity (CRS and neurotoxicity), off-target effects
Manufacturing/scalability	Faster, flexible, cost-effective, scalable (inspired by mRNA vaccine platforms)	Complex, expensive viral production pipelines
In vivo adaptability	Emerging potential for direct in vivo programming of CAR-T cells via injectable LNP-mRNA formulations	Limited; typically requires ex vivo manipulation
Clinical/preclinical examples	BCMA mRNA CAR-T (Descartes-08), Glioblastoma CAR+ cytokine, myasthenia gravis cases.	Well established for hematologic malignancies, challenges for solid tumors

**Table 3:** Comparison of CAR-T, CAR-NK, and TCR-T Cell Therapies.

Feature	CAR-T Cells	CAR-NK Cells	TCR-T Cells
Feasibility	Clinically mature with many FDA-approved products, typically autologous manufacturing	Moderate; can be derived from donors, cord blood, or cell lines, enabling “off-the-shelf” production	Moderate; requires patient- specific HLA matching and identification of suitable tumor antigens
Efficacy	Very high efficacy in hematologic cancers (e.g., B cell leukemias and lymphomas), however, limited success in solid tumors	Promising early clinical activity, particularly in hematologic cancers, efficacy in solid tumors still under evaluation	Demonstrated efficacy in selected solid tumors and hematologic cancers by targeting intracellular antigens
Key Advantages	Strong antigen-specific cytotoxicity, long in vivo persistence, well-validated clinical platform	Lower risk of cytokine release syndrome (CRS) and neurotoxicity, minimal GvHD risk, innate and CAR-mediated killing, scalable manufacturing	Ability to target intracellular tumor antigens via MHC presentation; broader antigen repertoire than CAR-based strategies
Key Limitations	High cost; complex manufacturing, severe toxicities (CRS, neurotoxicity), antigen escape and limited solid tumor efficacy	Shorter persistence and reduced in vivo expansion; limited clinical data compared with CAR-T, tumor trafficking challenges	HLA restriction limits patient eligibility, risk of off-target or cross-reactive toxicity, antigen escape, HLA downregulation, complex antigen discovery and validation

## Data Availability

No new data were generated or analyzed in this review. All data discussed are from published studies available through PubMed search.

## Abbreviations

AVVs	Adeno-associated viruses
B-ALL	B cell acute lymphoblastic leukemia
BCMA	B cell maturation antigen
BTLA	B and T lymphocyte attenuator receptor
CAR	Chimeric antigen receptor
CCR	Chemokine receptors
CRISPR	Clustered regularly interspaced short palindromic repeats
CRS	Cytokine release syndrome
CTLA-4	Cytotoxic T-lymphocyte associated protein 4
CXCR	C-X-C chemokine receptor
DNA	Deoxyribonucleic acid
FITC	Fluorescein isothiocyanate
GvHD	Graft-versus-host disease
IL	Interleukine
ITIM	Immunoreceptor tyrosine-based inhibitory motif
LIR-1	Leukocyte immunoglobulin-like receptor 1
LNP	Lipid nanoparticles
m1ψ	1-methyl pseudouridine
mRNA	Messenger RNA
NK	Natural killer
NHL	Non-Hodgkin lymphoma
PD1	Programmed cell death protein 1
R/R	Relapsed or refractory
ψ	Pseudouridine
TCR	T cell receptor
TIGIT	T cell immunoglobulin and ITIM domain
TME	Tumor microenvironment
TNF-α	Tumor necrosis factor alpha
UTR	Untranslated region
